# Molecular property prediction by contrastive learning with attention-guided positive sample selection

**DOI:** 10.1093/bioinformatics/btad258

**Published:** 2023-04-20

**Authors:** Jinxian Wang, Jihong Guan, Shuigeng Zhou

**Affiliations:** Shanghai Key Lab of Intelligent Information Processing, and School of Computer Science, Fudan University, 2005 Songhu Road, Shanghai 200438, China; Department of Computer Science and Technology, Tongji University, 4800 Cao'an Road, Shanghai 201804, China; Shanghai Key Lab of Intelligent Information Processing, and School of Computer Science, Fudan University, 2005 Songhu Road, Shanghai 200438, China

## Abstract

**Motivation:**

Predicting molecular properties is one of the fundamental problems in drug design and discovery. In recent years, self-supervised learning (SSL) has shown its promising performance in image recognition, natural language processing, and single-cell data analysis. Contrastive learning (CL) is a typical SSL method used to learn the features of data so that the trained model can more effectively distinguish the data. One important issue of CL is how to select positive samples for each training example, which will significantly impact the performance of CL.

**Results:**

In this article, we propose a new method for molecular property prediction (MPP) by ***C****ontrastive* ***L****earning with* ***A****ttention-guided* ***P****ositive-sample* ***S****election* (CLAPS). First, we generate positive samples for each training example based on an attention-guided selection scheme. Second, we employ a Transformer encoder to extract latent feature vectors and compute the contrastive loss aiming to distinguish positive and negative sample pairs. Finally, we use the trained encoder for predicting molecular properties. Experiments on various benchmark datasets show that our approach outperforms the state-of-the-art (SOTA) methods in most cases.

**Availability and implementation:**

The code is publicly available at https://github.com/wangjx22/CLAPS.

## 1 Introduction

MPP is to estimate the properties of molecules by a model trained on a set of molecules with known properties, which is one of the fundamental tasks in drug design and discovery, and can accelerates the process of designing and discovering molecules with desirable properties (David et al. 2020). With the development of machine learning and deep learning (DL), data-driven molecular representation learning has widely applied to MPP (Chithrananda et al. 2020; Fang et al. 2022a).

To predict molecular properties, we have to solve three problems: (i) Input molecule representations—describing molecules in a computable format such as SMILES strings or graphs; (ii) Feature extraction or feature learning—transforming a molecule to a feature vector; and (iii) Prediction—training a predictive model with a large number of labeled molecules represented as feature vectors. For molecule representation, currently, there are two widely used methods. One is a string-based molecular representation such as IUPAC, ECFP (Rogers and Hahn 2010), and SMILES (Weininger 1988). Molecules are linearized into strings of characters representing atoms and bonds. This is a traditional representation method in computational chemistry and biology. With string representations, we can utilize autoregressive models (Vaswani et al. 2017) to capture the chemical patterns hidden among atoms and bonds. The other is graph-based molecular representation (Hu et al. 2019), i.e. a molecule is represented as a graph, where nodes and edges represent atoms and chemical bonds, respectively. As for feature extraction and prediction, DL, and deep neural network (DNN) models have been become the mainstream in bioinformatics. There are two types of models for property prediction: classification models for categorical properties (e.g. whether or not a molecule is toxic) and regression models for numerical properties (e.g. the water-solubility value of a molecule).

In recent years, *self-supervised learning* (SSL) has shown its great success in artificial intelligence (AI) tasks such as image recognition (Ulyanov et al. 2018), image denoising (Xie et al. 2020), and single-cell data analysis (Batson and Royer 2019). In addition, it has also made remarkable progress in representation learning for different types of data, including texts (Devlin et al. 2018; Wu et al. 2019), images (Chen et al. 2020; Hassani and Khasahmadi 2020), and graphs (Narayanan et al. 2017). A typical SSL paradigm is *contrastive learning* (CL), where unlabeled data points are juxtaposed against each other to teach a model which pairs are similar and which pairs are different. CL has achieves promising performance in various AI and bioinformatics tasks (Hu et al. 2021; Wang et al. 2021). One of key problems of CL is how to construct new positive samples for each training example.

**Figure 1.**
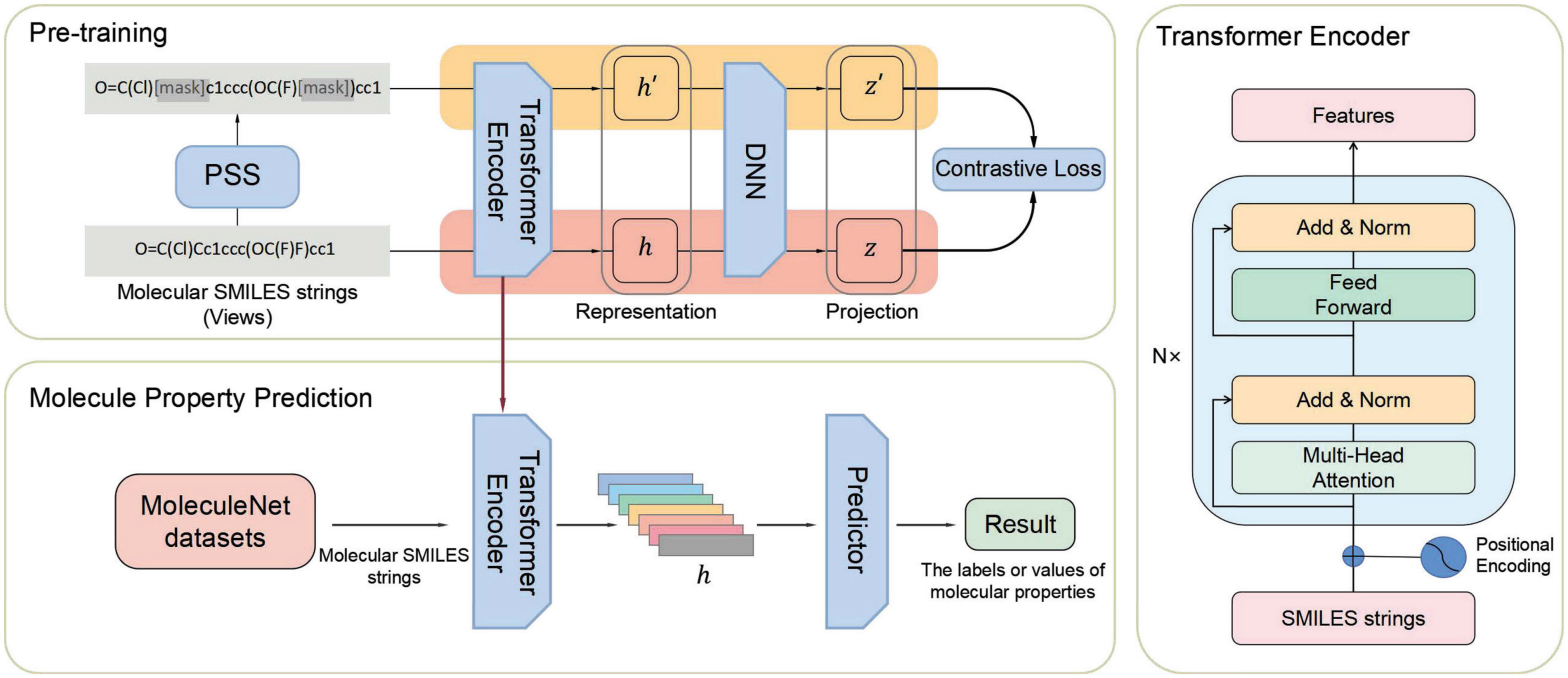
The framework of our method CLAPS. Here, The top-left part is the pretraining module that consists of three major components: PSS, transformer encoder, and contrastive loss, the bottom-left part is the property prediction module that consists of two major components: the trained transformer encoder and a predictor. The right part shows the structure of a transformer encoder.

In this article, to boost the prediction of molecular properties, we propose a new method (the framework is shown in Fig. 1) called CLAPS, which uses CL with attention-guided positive sample selection (PSS) to learn molecule representations that are invariant and robust enough, while keeping the latent information in molecules. Concretely, we exploit a large number unlabeled molecules as input to pretrain the model, which is used for MPP. The PSS module in our method consists of a self-attention network and a masking strategy. We first design a trainable multilayer multihead self-attention network to generate a weight matrix, and then mask the corresponding positions in the weight matrix according to the masking strategy to generate samples. Each generated sample and its original sample are then input into the CL framework for model pretraining, and a Transformer encoder suitable for SMILES strings is adopted to extract latent feature vectors. Finally, a DNN is used for prediction, during which the contrastive loss is employed to make the model distinguish positive and negative sample pairs well to extract better molecular representations for property prediction. CLAPS can also be applied to handling other types of data such as images and texts.

In summary, the contributions of this article are as follows: (i) We propose a new method that employs CLAPS to promote MPP. (ii) We design an attention-guided sample generation module to select positive samples for CL. (iii) We conduct extensive experiments to evaluate CLAPS, and experimental results validate its effectiveness and advantage in MPP.

## 2 Related work

*DL* has achieved great success in various areas, which inspires its application in MPP. Many works of MPP apply DNNs to learning molecular representations based on molecular SMILES strings or graphs (Wang et al. 2022). Molecular SMILES strings describe the compositions and chemical structures of molecules. A number of DNN models such as RNN, LSTM, and Transformer have been used to build MPP models. Recently, *SSL*-based models like Bert have been applied to learning meaningful representations from large amounts of unlabeled data for bioinformatics tasks (Chithrananda et al. 2020).

Conceptually, SSL is a machine learning paradigm that enables to train deep models with unlabeled data, overcoming the problem of lack of annotated labels. So, it can learn representations from unlabeled data itself when no labeled data are available. SSL is usually used as a pretraining process on unlabeled data, after which a limited number of labeled data are adopted to fine-tune the pretrained deep models for downstream tasks. Existing SSL methods can be further divided into predictive learning based and *CL* based (Xie et al. 2022).

Hu et al. (2019) is a predictive learning model based on graphs for predicting molecular properties, which consists of two main modules: context prediction and attribute masking prediction. It can learn both local and global representations simultaneously by pretraining a SSL model to capture the structural patterns of graphs. String-based models are simpler and more efficient compared with graph-based models (Honda et al. 2019). Transformer (Vaswani et al. 2017; Devlin et al. 2018) has become a general architecture for SSL of strings for many tasks in natural language processing and biological data mining, where the transformer is usually first pretrained, and the encoder is then fine-tuned for specific tasks to improve the model’s performance. For example, SMILES Transformer (ST; Honda et al. 2019) and ChemBERTa (Chithrananda et al. 2020) both establish a molecular string-based model based on SMILES strings, which is pretrained under the transformer-based framework. Additionally, transformer attention-based methods also play an important role in other studies. Chemformer (Irwin et al. 2022), a transformer-based model, is applied to discriminative cheminformatics tasks. And the sAMPpred-GATThe Chemformer (Yan et al. 2023) is a model using graph attention network to predict Antimicrobial peptides. However, according to experimental results, this model seems not robust enough.

CL aims to discriminate jointly sampled view pairs (e.g. two views of the same instance) from independently sampled view pairs (e.g. views of different instances), it can overcome the limitations of the Transformer framework to some degree. In particular, the positive view pairs are usually generated from the same instance and the negative view pairs are usually generated from different instances. We can obtain multiple views from each instance by applying different transformations such as masking and dropping for molecules. CL has been proved effective in various tasks (Chen et al. 2020; Hassani and Khasahmadi 2020).

According to the construction method of new samples, CL methods can be further divided into two types. One is to construct negative samples. For example, MoCo (He et al. 2020) first establishes a large negative sample queue, then uses a first-in, first-out strategy to iteratively update the negative samples in the queue. However, only a small fraction of the oldest negative samples in the queue are updated and we cannot continuously track the changes of feature representations during the iteration process. The newly proposed AdCo (Hu et al. 2021) model improves that by using a generative network to construct a set of negative samples. But, its convergence is not guaranteed theoretically because of a separately trained model. Currently, there is no CL that constructs negative samples for MPP.

The other is to construct positive samples. GraphCL (You et al. 2020) proposes a *graph contrastive learning* (GCL) framework for learning unsupervised representations of molecular graphs. It designs four types of graph augmentations including node dropping, edge perturbation, attribute masking, and subgraphs to construct positive samples. Similar to GraphCL, Molclr (Wang et al. 2021), a GCL method, constructs positive samples by three novel molecule graph augmentations: atom masking, bond deletion, and subgraph removal. Then, a contrastive estimator is utilized to maximize the agreement of different graph augmentations from the same molecule. On the contrary, Fang et al. (2022b) pointed out that most existing augmentation approaches (e.g. node dropping and edge perturbation) violate the chemical semantic inside molecules and ignore the influence of fundamental knowledge on graph semantics, thus they add new nodes and edges into molecules under the guidance of Chemical Element Knowledge Graph to construct positive samples.

The representations generated by CL focus on the information sharing between samples (Tian et al. 2020), and the sample augmentation scheme is an important component in CL. A study (van den Oord et al. 2018) shows that sample selection depends on the downstream tasks. There may be optimal strategies corresponding to different downstream tasks. And CL also has the robust problem of unstable representation.

## 3 Materials

On the one hand, we use molecules from the ZINC15 database (Sterling and Irwin 2015) for pretraining. On the other hand, we predict properties of molecules in benchmark datasets from MoleculeNet (Wu et al. 2018).

### 3.1 Molecule data for pretraining

The ZINC15 database utilizes third-party databases and libraries such as ChEMBL (Gaulton et al. 2012), HMDB (Wishart et al. 2022) and DrugBank (Wishart et al. 2018) to annotate the compounds. It also includes commercially available biomolecular drug-like compounds such as natural products, metabolites, and FDA-approved drugs. We used 276 003 molecules, reported or inferred to be active at 10 μM or higher concentrations in direct binding assays, to pretrain an Encoder.

### 3.2 Molecule property data

We evaluate our model CLAPS and compare it with SOTA MPP methods on various benchmark datasets (the data details are presented in Table 1). These datasets differ from each other in their respective domains, task types, and sizes, which can be grouped into the following three categories:

**Table 1.** Summary information of the MoleculeNet benchmark datasets.

Dataset type	Dataset	Tasks	Task type^a^	Molecules	Evaluation indicators	Describe
Physical chemistry	ESOL	1	R	1128	RMSE	Water solubility
	FreeSolv	1	R	643	RMSE	Hydrogen free energy
	Lipo	1	R	4200	RMSE	Octanol/water distribution ratio, coefficient (logD)
Biophysics	HIV	1	C	41 913	ROC AUC	The ability to suppress HIV replication
	BACE	1	C	1522	ROC AUC	Binding results of human BACE-1 inhibitors
Physiology	BBBP	1	C	2053	ROC AUC	Blood-brain barrier penetration
	Tox21	12	C	8014	ROC AUC	Toxicity
	SIDER	27	C	1427	ROC AUC	Adverse drug reactions to the 27 systemic organs
	ClinTox	2	C	2053	ROC AUC	Clinical trial toxicity and FDA approval status

a R and C in the task type column indicate the regression and classification tasks, respectively.

- Physical chemistry datasets: ESOL, FreeSolv, and Lipo.
- Biophysical datasets: HIV and BACE.
- Physiological datasets: BBBP, Tox21, SIDER, and ClinTox.

Here, ESOL, FreeSolv, and Lipo are regression tasks where the model outputs numerical property values, and the others are classification tasks where the model outputs categorical property labels. Particularly, Tox21, SIDER, and ClinTox are multilabel classification tasks. Additionally, each dataset is split into the training/validation/test subsets, following an 8/1/1 ratio. The training sets are used for training models, while the validation sets for tuning hyperparameters, and the test sets for model evaluation. Notably, we split structurally different molecules into different subsets based on their 2D structures in terms of scaffold (Bemis and Murcko 1996). For example, benzene, naphthalene, and anthracene are all single-ring systems that will be divided into the same subset, and the others will be another subset. Therefore, the scaffold-based splitting method offers a greater challenge for the model than randomly splitting. For each molecule, the input of our CLAPS method is its SMILES string. For example, for a molecule from the Clintox dataset, its SMILES string “C[NH+]1CCC[C@H]1c2cccnc2” is used as the input, our method will output two labels: “1” and “0.” More detailed description of each dataset is in the Supplementary File S1.

## 4 Methods

### 4.1 Framework

The framework of our method CLAPS is shown in Fig. 1. CLAPS is based on CL with an attention-guided PSS mechanism. We use CL to learn robust molecular representations for accurate property prediction and an attention-based mechanism to generate proper positive samples for CL. CLAPS consists of two modules (or stages): pretraining (the top-left part of Fig. 1) and prediction (the bottom-left part of Fig. 1). In the pretraining stage, there are three major components: the *PSS* component for positive sample generation, the *transformer encoder* for molecule representation learning and a *contrastive loss* component to constrain the learning of molecular representations. PSS first takes SMILES strings of molecules as input to generate new positive samples. Then, we use the generated positive samples and the corresponding original samples into the contrastive loss component in pairs to obtain the projections after encoding by the encoder and a DNN. Finally, the contrastive loss is calculated for pretraining. In the prediction stage, molecules from MoleculeNet are encoded by the *transformer encoder* trained above to vector representations, which are predicted by a predictor consisting of three fully connected layers to get the final property values.

In summary, our goal is to pretrain the encoder to generate informative molecular embeddings, while ensuring that the SMILES string embeddings are robust and transferable across benchmark datasets presented in Table 1.

### 4.2 Generating positive samples

**Figure 2.**
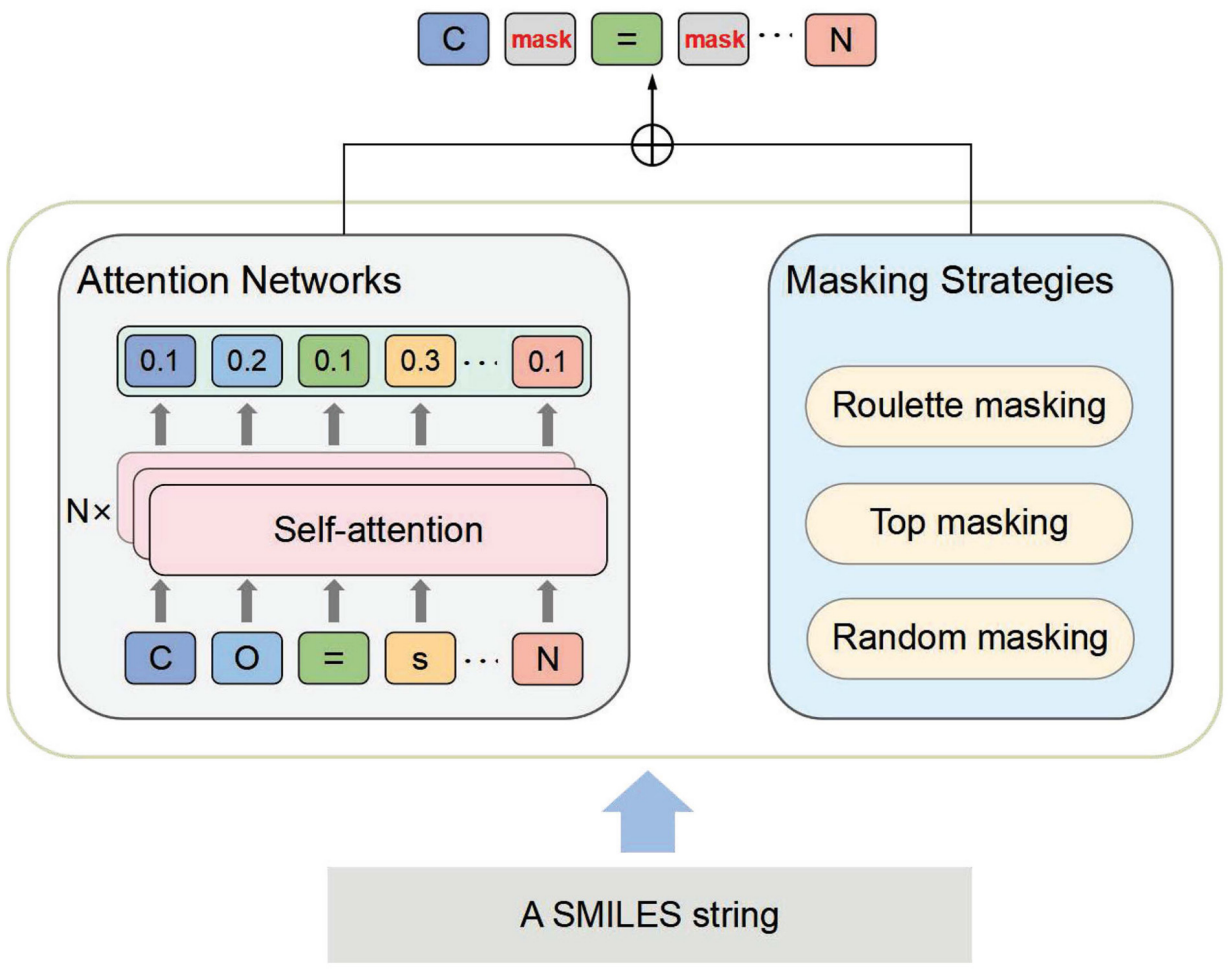
An illustrative example of PSS.

The quality of generated samples has a great impact on the performance of SSL. In this article, our PSS module includes two parts: self-attention and masking as illustrated in Fig. 2.

SMILES is a string-based chemical language with a simple vocabulary (atom and bond symbols) and some grammar rules. We input SMILES strings of molecules to a trainable multilayer multi-head self-attention network to obtain the attention weight matrix. Concretely, we first obtain the SMILES strings of molecules and traverse all character types in the pretraining set, recording all occurrences of characters (e.g. “c,” “cl,” and “S” element characters) into the dictionary. Then, for a molecular SMILES string, we replace its characters with their IDs in the dictionary, and embedding each character to a vector by randomly initialization, where the vector’s element values are randomly set by following the normal distribution N(0,1). So, the feature of each molecular can be expressed as $S\in R^{C\times d}$, where *C* is the fixed string length, and *d* is the vector dimension of characters. Furthermore, in order to get the position information of the molecular string, we use sine-cosine encoding to obtain a set of position vectors $P\in R^{C\times d}$. We employ the special character “pad” to fill in the part of molecular string whose length does not reach *C*. Finally, we get the $X\in R^{C\times d}$ by adding $P\in R^{C\times d}$ to $S\in R^{C\times d}$, and take *X* as the input of a multihead self-attention network. Thus, we get an attention (weight) matrix *Att*_*i*_ normalized by softmax from the *i*th head self-attention network as follows: where $W_{i}^{q}$ and $W_{i}^{k}$ are parameterized matrices, *head* indicates the number of heads in the multi-head self-attention network, which is set to 3 in our article. Finally, the attention weight vector is obtained by first summing the attention matrices, i.e. $\sum_{i}^{\mathrm{head}}Att_{i}\in R^{C\times C}$, then summing the elements of each row of the resulting matrix.


(1) $$Att_{i}=\text{softmax}\left(\frac{XW_{i}^{q}\times {\left(XW_{i}^{k}\right)}^{T}}{\sqrt{d}}\right),i=1,2,\ldots ,\mathrm{head}$$

With the weight vector above, we mask the characters in the SMILES string according to the corresponding weights and generate a new positive sample by different masking strategies. We define three different weight-masking strategies (as shown Fig. 2) for SMILES strings as follows:

- *Roulette masking*: After calculating the weight vector by the self-attention mechanism, the selection probability of each character is set proportional to its weight. So the higher the weight, the more likely to be masked. Concretely, the probability $p_{i}$ of the *i*th character is $\frac{f_{i}}{\sum f_{i}}$ where $f_{i}$ is the occurrences of the *i*th character in the processed SMILES string. Randomly generate a value *r* between 0 and 1, if $p_{1}+p_{2}+\cdots +p_{i-1}<r\leq p_{1}+p_{2}+\cdots +p_{i}$, then select the *i*th character to mask. This method not only ensures that characters with larger weights are more probably to be masked, but also takes into account the global information.
- *Top masking*: The characters with the top weights are masked. In our article, we mask out the characters with the top 10%, 25%, and 50% weights (denoted by top-10%, top-25%, and top-50%, respectively) to generate positive samples.
- *Random masking*: Randomly masking the characters in the SMILES string. Concretely, we randomly mask out 10%, 25%, and 50% (denoted by random-10%, random-25%, and random-50%, respectively) the characters to generate positive samples.

### 4.3 Transformer encoder and DNN

As we know that the CL framework allows various choices of the network architecture. Here, we adopt the commonly used transformer encoder to extract representation vectors *h* and $h^{\prime }$ of augmented data examples, and the encoder that consists of three layers. Each layer of the transformer encoder is composed of two networks: a four-head self-attention network and a position-wise fully connected feed-forward network. And, we use a residual connection and layer normalization after each of the two networks. The output of each network is LayerNorm(x+sublayer(x)) where the sublayer(x) is the function implemented by neural network.

We first get the input $X\in R^{C\times d}$ of the transformer encoder, which are the embeddings of normal SMILES strings or masked SMILES strings. The output of each transformer encoder layer is as follows: where $W_{i}^{v}$ and $W^{O}$ are parameterized matrices, the *h* is the number of heads. After the attention network, a fully connected feed-forward network consisting of two linear transformations with a Rectified Linear Unit activation is applied.


(2) $$X_{\mathrm{att}}=\mathrm{Concat}(Att_{1}(XW_{1}^{v}),\ldots ,Att_{h}(XW_{h}^{v}))W^{O}$$

In summary, we build a transformer encoder with three layers. Each transformer encoder layer has a four-head self-attention network and a feed-forward network.

Then, a DNN consisting of two-layer perceptron without bias maps representations to the projection space: where $W_{1}$ and $W_{2}$ are learnable weight parameters, *h* is the representation. Finally, the contrastive loss is evaluated on *z*.


(3) $$z=W_{1}R\mathrm{eLU}(W_{2}h)$$

### 4.4 Contrastive loss

The goal of CL is to learn the similarity and difference among the samples. We randomly get a minibatch of *N* molecules and generate their positive samples by the method in Section 4.2, resulting in 2*N* molecule samples. We aim to maximize the similarity between the projection vectors of each positive pair, and minimize the similarity between each sample and any of the other 2(N−1) negative projection vectors in the minibatch.

In the representation space, we want positive pairs to be as close as possible and negative pairs to be as far as possible. We define a suitable loss function to achieve this. Before introducing the loss function, we need a function to measure the distance or similarity between two vectors in the projection space. Generally, a similarity function is used. Specifically, the similarity function adopts point product or cosine similarity after $L_{2}$ regularization of the representation vectors $z_{i}$ and $z_{j}$. That is,


(4) $$Sim_{\left(z_{i},z_{j}\right)}=\frac{z_{i}^{T}z_{j}}{\left||z_{i}||||z_{j}|\right|}.\;$$

We use the loss function of the CL framework SimCLR (Chen et al. 2020), which uses a normalized temperature-scale cross-entropy loss (NT-Xent loss). Given the batch size *N*, the loss function of positive pairs can be evaluated as follows: where $E_{\left[k\neq i\right]}$ is an indicator function whose value is 1 if k≠i, otherwise is 0. *T* is a temperature parameter. The final loss is the average of the loss sum of all sample pairs in each batch as follows:


(5) $$\ell_{i,j}=-\text{log}\;\frac{\;\text{exp}(Sim_{\left(z_{i},z_{j}\right)}/T)}{\sum_{k=1}^{2N}E_{\left[k\neq i\right]}\;\text{exp}(Sim_{\left(z_{i},z_{k}\right)}/T)}$$

(6) $$L=\frac{1}{2N}\sum_{k=1}^{N}[\ell (2k-1,2k)+\ell (2k,2k-1)].$$

### 4.5 Property prediction loss

We use the pretrained encoder to embed the molecules and get the latent features. Then, we predict the molecular properties by a predictor that consists of three FC layers. In our experiments, the best prediction performance is obtained when the number of neurons in the three FC layers is [4096, 512 64], respectively. The loss of classification is *binary cross entropy*: where *t* is the target label, *o* is the predicted label that is first processed by the sigmoid function, *n* indicates the number of total labels.


(7) $$\mathrm{BCE}(o,t)=-1/n\sum_{i}\left(t[i]\times \text{log}(o[i])+(1-t[i])\times \text{log}(1-o[i])\right)$$

Meanwhile, we utilize the *root mean squared error* (RMSE) for the regression task, i.e.: where *Y* is the real value and $\hat{Y}$ is the predicted value.


(8) $$\mathrm{RMSE}(Y,\hat{Y})=\sqrt{1/n\sum_{i=1}^{n}{\left(Y_{i}-\hat{Y_{i}}\right)}^{2}}$$

### 4.6 Performance metrics

As suggested by MoleculeNet, the area under the ROC characteristic curve (ROC-AUC) is used as the performance metric for classification on datasets BBBP, Tox21, SIDER, ClinTox, HIV, and BACE. ROC-AUC is used to evaluate the performance of multilabel binary classification tasks, and the higher the ROC-AUC is, the better the performance is. On the three regression datasets ESOL, FreeSolv, and Lipo, we use RMSE to evaluate the performance, and the lower RMSE is, the better the performance is.

## 5 Results

### 5.1 Baselines and experimental settings

We comprehensively evaluate our method and compare it against eight state-of-the-art (SOTA) self-supervised methods based on string or graph molecular representations. Four string-based models are as follows:

- ChemBERTa (Chithrananda et al. 2020): A model with a transformer architecture for predicting molecular property, it uses the masked language-modeling pretraining task to provide improvement on predictive power for models on downstream tasks from MoleculeNet.
- SMILES Transformer (Honda et al. 2019): ST learns molecular fingerprints through unsupervised pretraining of sequence to sequence language models.
- RNNS2S (Xu et al. 2017): A text-based pretraining model that uses RNN Seq2seq network for model architecture construction.
- ECFP4 (Rogers and Hahn 2010): This method uses manually generated drug fingerprints with prior knowledge. It hashes multiscale substructures into integers and produces a fixed-length binary vector, where 1 indicates the presence of the assigned substructure and 0 indicates its absence.

The four graph-based models are:

- MolCLR (Wang et al. 2021): It is developed upon the CL framework. Latent representations of positive augmented molecule graph pairs are contrasted against representations of negative pairs.
- GraphCL (You et al. 2020): It employs CL with augmentations for Graph Neural Network (GNN) pretraining to address the challenge of data heterogeneity in graphs.
- GROVER (Rong et al. 2020): It can learn rich structural and semantic information of molecules from enormous unlabeled molecules. Rather, to encode such complex information, GROVER integrates message passing networks into the transformer-style architecture to deliver a class of more expressive encoders of molecules.
- GraphConv (Duvenaud et al. 2015): It consists of convolutional neural networks, allows end-to-end learning of prediction pipeline with arbitrary size and shape graphs, and predicts molecular property without pretraining.

The dropout is set to 0.2 at both the pretraining stage and the prediction stage, but the learning rate is set to 1e−3 at the pretraining stage and le−4 at the prediction stage. The number of pretraining epochs is set to 30. All experiments used the Adam optimizer and are conducted on NVIDIA RTX3090 GPUs. Major parameter settings of the pretraining stage are summarized in Table 2.

**Table 2.** Parameter settings in CLAPS.^a^

Parameter	Value
Weight masking ratio	10%, **25%**, 50%
Sample selection strategy	Top masking; **Roulette masking**;
	Random masking
Batch size	500, 1000, **1500**
DNN	[1024, 512], **[2048, 512]**, [2048, 1024], [4096, 512]
Learning rate	$10^{-2}$ , **10^−3^**, $10^{-4}$, $10^{-5}$
Dropout	No dropout, 0.1, **0.2**, 0.3, 0.4, 0.5

a Default values are in bold black.

### 5.2 Performance evaluation and comparison

We present the performance results of MPP on the classification and regression datasets in Table 3. From the results of classification, we can see that (i) Our method achieves the best performance on three of the six classification datasets, demonstrating the effectiveness of our method by CLAPS. (ii) The roulette masking strategy performs better than the other two masking strategies on the 6 classification datasets except for ClinTox. (iii) CLAPS achieves an overall performance improvement of 7.3% in terms of the average ROC-AUC compared with the SOTA result from the MolCLR method.

**Table 3.** Performance comparison on classification and regression datasets.^a^

Model	Method	HIV↑	BACE↑	BBBP↑	Tox21↑	SIDER↑	ClinTox↑	Average	ESOL↓	FreeSolv↓	Lipo↓	Average
Ours	CLAPS (Roulette)	0.779	0.723	**0.962**	0.733	**0.751**	0.976	**0.821**	**0.547**	**0.907**	0.818	**0.757**
	CLAPS (top25%)	0.769	0.723	0.945	0.723	0.748	0.956	0.811	0.662	1.091	0.954	0.902
	CLAPS (random25%)	0.510	0.675	0.904	0.623	0.734	**0.985**	0.740	0.731	1.314	0.881	0.975
String-based	ChemBERTa	0.622	^b^	0.643	0.728	^b^	0.733	0.682	^b^	^b^	**^a^**	^b^
	ST	0.683	0.719	0.900	0.706	0.559	0.963	0.755	1.144	2.246	1.169	1.519
	ECFP4+MLP	0.679	0.769	0.760	0.616	0.588	0.515	0.655	1.741	3.043	1.090	1.958
	RNNS2S+MLP	0.682	0.717	0.884	0.702	0.558	0.904	0.741	1.317	2.987	1.219	1.841
Graph-based	MolCLR	0.778	0.788	0.738	**0.747**	0.669	0.867	0.765	1.160	2.390	**0.780**	1.443
	GraphCL	**0.784**	0.753	0.696	0.738	0.605	0.759	0.723	^b^	^b^	**^b^**	^b^
	GROVER	0.625	**0.826**	0.700	0.743	0.648	0.812	0.726	0.983	2.176	0.817	1.325
	GraphConv	0.723	0.744	0.795	0.687	0.557	0.936	0.740	1.673	3.476	1.062	2.070

a The SOTA results are shown in bold.

b The result is unavailable in the original paper.

For the regression results, we also have the following observations: (i) Our method achieves the best performance on two of the three regression datasets, demonstrating its effectiveness and advantage. (ii) The roulette masking strategy performs better than the other two masking strategies on the three regression datasets. (iii) The average RMSE of CLAPS is 42.8% lower than that of GraphCL, a SOTA graph-based method.

Generally, our experiments on multiple tasks show that CLAPS is superior or comparable to the SOTA approaches. Concretely, the results in Table 3 show that our method CLAPS with random 25% can achieve better results on multiple datasets than existing string-based methods, which shows that CL can effectively improve model performance on string-based data. In addition, the performance of the roulette and top masking strategies is better than random masking, which indicates that our PSS mechanism can help improve the performance of CL. Furthermore, compared with the methods based on GCL with PSS (e.g. MolCLR and GraphCL), our method is significantly superior on multiple datasets. Overall, our method is effective in molecular feature extraction for property prediction.

### 5.3 Ablation study

Here, we conduct ablation experiments on the classification and regression datasets above to explore the influence of different probabilities of masking on CLAPS performance. There are three masking probabilities for comparison: 10%, 25%, and 50%.

**Figure 3.**
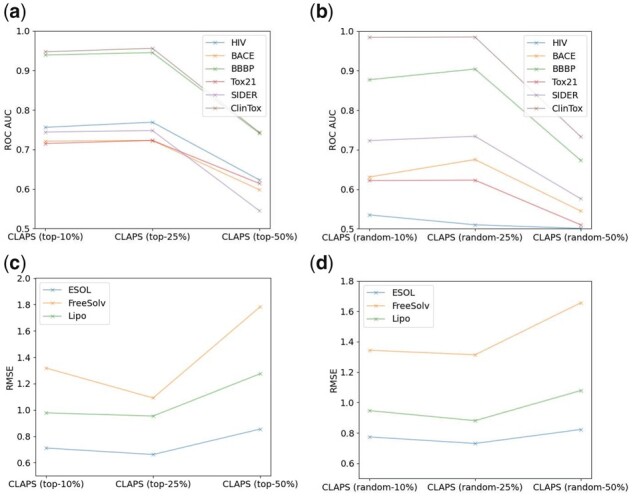
(a) The performance comparison between top masking strategies on classification datasets. (b) The performance comparison between random masking strategies on classification datasets. (c) The performance comparison between top masking strategies on regression datasets. (d) The performance comparison between random masking strategies on regression datasets.

Figure 3a and b shows the performance comparison between top masking and random masking on six classification datasets. Both strategies are found to achieve the best performance when the masking probability is 25%. Figure 3c and d shows the performance comparison between top masking and random masking on the three regression datasets. Similar to the results of classification datasets in Fig. 3a and b, we can see that the two strategies achieve the best performance when the masking probability is 25%.

**Figure 4.**
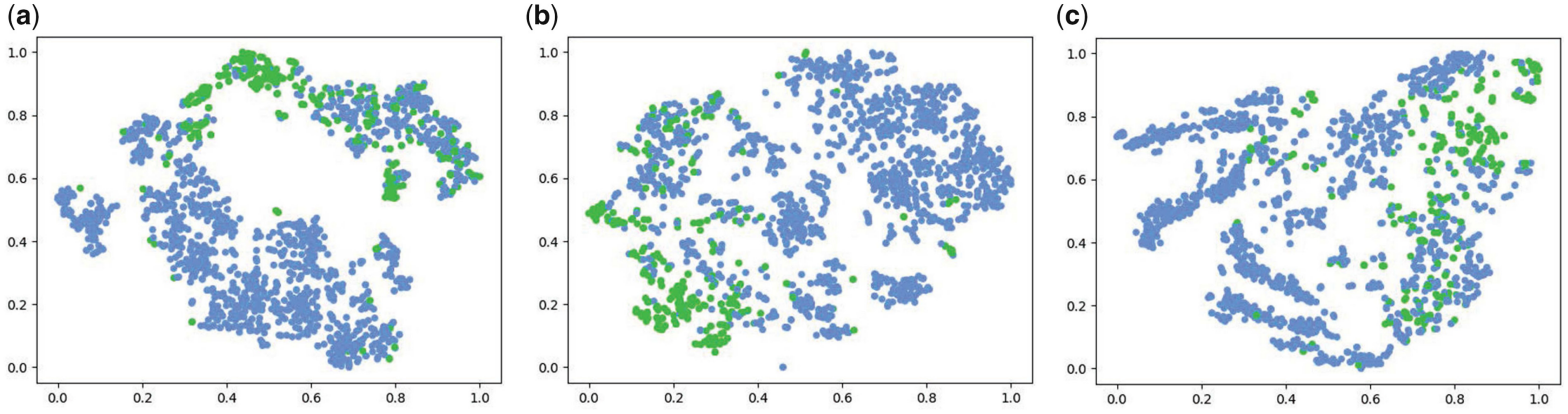
t-distributed Stochastic Neighbor Embedding (t-SNE) visualization of BBBP. The blue points belong to the active class and the green points belong to the inactive class. (a) Roulette, (b) Top-25%, and (c) Random-25%.

In addition, to explore why CLAPS with roulette masking achieves good predictive performance, we first embed the training set of BBBP to a latent space, then we reduce the dimensionality of the embeddings by a classical dimensionality reduction method t-SNE (set the learning_rate to 600, the other parameters are default; Van der Maaten and Hinton 2008). Finally, we visualize the data after normalization. The results are shown in Fig. 4, where (Fig. 4a–c) correspond to the results of the roulette masking, top masking, and random masking, respectively. We can clearly see that in Fig. 4a, data points of active class are separated better from the inactive class points than that in Fig. 4b, whereas in Fig. 4c data points of the two classes are mixed. This explains why the roulette masking strategy outperforms the other two masking strategies.

**Figure 5.**
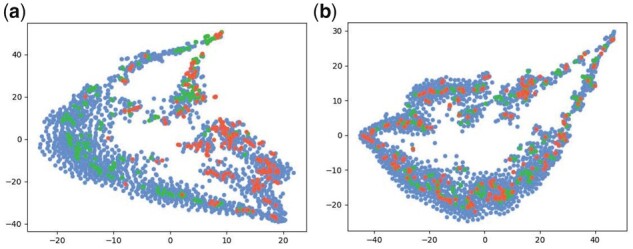
t-SNE visualization of BBBP with (a) scaffold-splitting and (b) random-splitting. The blue points belong to the train set, the green points belong to the valid set and the red points belong to the test set.

Moreover, we check the effect of similarity of subsets on the prediction results. We first split the BBBP dataset by scaffold-based and random-based methods, and embed the data. Then, we perform dimensionality reduction and visualization over the embedded data by t-SNE. The results are shown Fig. 5a and b, from which we can see that the training and testing datasets obtained by scaffold-splitting are less overlapped in distribution than the training and testing datasets obtained by random-splitting. And CLAPS achieves a 0.977 AUC-ROC on the random-split BBBP dataset, which is better than that on the scaffold-split BBBP dataset. This result is consistent with our expectation.

## 6 Conclusion

In this article, we propose a new method called CLAPS for MPP by CL with attention-guided PSS. CLAPS first pretrains the model on a unlabeled molecular dataset via CL to push the negative pairs away in the feature space, and separate the samples that may overlap, which thus benefits the training of the model. The key of CL is how to generate new samples from the same instance. We use an attention-guided, instead of stochastic-based or heuristic-based, mechanism to generate new samples automatically by the PSS module, where the weight of each character of a SMILES string is obtained by attention, then generates new samples by top masking strategy or roulette masking strategy. Our attention-guided sample generation method can effectively avoid the problems with existing methods: (i) important character information loss in stochastic-based methods; (ii) limited model robustness of heuristic-based methods. Furthermore, the new samples may be significantly different from the original samples, and the loss of CL will enlarge the distance of positive pairs in the feature space. In our point of view, there seems adversarial learning between the PSS module and the CL module, which enables CLAPS to reach a stable state in pretraining.

To the best of our knowledge, CLAPS is the first work to leverage CL to molecular data of SMILES string form to predict molecular properties. In addition, we apply text-based Transformer to SMILES strings to learn better representations. Experimental results on the MolecularNet benchmark datasets show that our method outperforms the SOTA string-based SSL methods, and is comparable to the SOTA graph-based methods.

String-based representations can be extended to cope with other biological data such as gene and protein sequences. Using the transformations produced by attention-guided perturbations can generate meaningful samples. One limitation of our method is that it uses pure character embedding while ignoring meaningful functional data. Furthermore, leveraging prior knowledge may have potential for performance improvement.

For the future work, we will try to improve the model interpretability and prediction performance by (i) integrating attention-guided sample selection with downstream tasks to further improve performance, (ii) predicting molecule properties by combining our method with graph-based molecular models, (iii) training the models with larger batch sizes on more powerful servers, and (iv) exploring causal constraints to CL to improve the interpretability of MPP.

## Supplementary Material

btad258_Supplementary_Data
