# Supplementary material for: Molecular property prediction by contrastive learning with attention-guided positive sample selection
Source: Bioinformatics. 2023 Apr 20;39(5):btad258. doi: 10.1093/bioinformatics/btad258 (PMC10188298; doi:10.1093/bioinformatics/btad258)
Supplement: btad258_Supplementary_Data [file btad258_supplementary_data.docx]

Content

1. Supplementary descriptions of MoleculeNet datasets

1. Supplementary descriptions of MoleculeNet datasets

BBBP: The Blood–brain barrier penetration (BBBP) dataset comes from a recent study (PMID: 22612593) on the modeling and prediction of the barrier permeability. As a membrane separating circulating blood and brain extracellular fluid, the blood–brain barrier blocks most drugs, hormones and neurotransmitters. Thus penetration of the barrier forms a long-standing issue in development of drugs targeting central nervous system. This dataset includes over 2,000 compounds on their permeability properties and the label is binary representation of permeability/non permeability.

BACE: The BACE dataset provides quantitative (IC50) and qualitative (binary label) binding results for a set of inhibitors of human β-secretase 1 (BACE-1) (PMID: 27689393). All data are experimental values reported in scientific literature over the past decade, some with detailed crystal structures available. Which have 1,522 compounds and the label is binary label of inhibitor.

HIV: The HIV dataset was introduced by the Drug Therapeutics Program (DTP) AIDS Antiviral Screen, which tested the ability to inhibit HIV replication for over 40,000 compounds (http://wiki.nci.nih.gov/display/NCIDTPdata/AIDS+Antiviral+Screen+Data). Screening results were evaluated and placed into three categories: confirmed inactive (CI), confirmed active (CA) and confirmed moderately active (CM). We further combine the latter two labels, making it a classification task between inactive (CI) and active (CA and CM). And the label is binary label of filter result: 1 (CA/CM) and 0 (CI).

Tox21: The “Toxicology in the 21st Century” (Tox21) initiative created a public database measuring toxicity of compounds, which has been used in the 2014 Tox21 Data Challenge (http://tripod.nih.gov/tox21/challenge/). This dataset contains qualitative toxicity measurements for 8,014 compounds on 12 different targets, including nuclear receptors and stress response pathways.

SIDER: The Side Effect Resource (SIDER) is a database of marketed drugs and adverse drug reactions (ADR) (PMID: 26481350). The version of the SIDER dataset in DeepChem has grouped drug side-effects into 27 system organ classes following MedDRA (http://www.meddra.org/) classifications measured for 1,427 approved drugs (following previous usage).

ClinTox: The ClinTox dataset, introduced as part of this work, compares drugs approved by the FDA and drugs that have failed clinical trials for toxicity reasons (PMID: 27642066). The dataset includes two classification tasks for 1,491 drug compounds with known chemical structures: (1) clinical trial toxicity (or absence of toxicity) and (2) FDA approval status. List of FDA-approved drugs are compiled from the SWEETLEAD database (PMID: 24223973), and list of drugs that failed clinical trials for toxicity reasons are compiled from the Aggregate Analysis of ClinicalTrials.gov (AACT) database (http://www.ctti-clinicaltrials.org/aact-database).

ESOL: ESOL is a small dataset consisting of water solubility data for 1,128 compounds (PMID: 15154768). The dataset has been used to train models that estimate solubility directly from chemical structures (as encoded in SMILES strings). Note that these structures don't include 3D coordinates, since solubility is a property of a molecule and not of its particular conformers.

FreeSolv: The Free Solvation Database (FreeSolv) provides experimental and calculated hydration free energy of small molecules in water (PMID: 24928188). The calculated values are derived from alchemical free energy calculations using molecular dynamics simulations. We include the experimental values in the benchmark collection, and use calculated values for comparison.

Lipo: Lipo is an important feature of drug molecules that affects both membrane permeability and solubility. This dataset, curated from ChEMBL database, provides experimental results of octanol/water distribution coefficient of 4,200 compounds.
